# Healthcare Professional Perspectives on Digital Health‐Related Quality‐of‐Life Assessment in Paediatric Radiation Therapy: A Qualitative Study

**DOI:** 10.1002/jmrs.880

**Published:** 2025-04-28

**Authors:** Mikaela Doig, Andrew Cunningham, Victoria Bedford, Hien Le, Matthew O'Connor, Eva Bezak, Nayana Parange, Amanda Hutchinson, Peter Gorayski, Michala Short

**Affiliations:** ^1^ UniSA Allied Health and Human Performance University of South Australia Adelaide South Australia Australia; ^2^ UniSA STEM University of South Australia Adelaide South Australia Australia; ^3^ Cancer Voices South Australia Adelaide South Australia Australia; ^4^ Department of Radiation Oncology Royal Adelaide Hospital Adelaide South Australia Australia; ^5^ Australian Bragg Centre for Proton Therapy and Research Adelaide South Australia Australia; ^6^ Michael Rice Centre for Haematology and Oncology Women's and Children's Hospital Adelaide South Australia Australia; ^7^ UniSA Justice and Society University of South Australia Adelaide South Australia Australia

**Keywords:** cancer survivors, implementation science, patient‐reported outcome measures, quality of life, radiation oncology

## Abstract

**Introduction:**

Health‐related quality of life (HRQoL) is not routinely evaluated using patient‐reported outcome measures (PROMs) in paediatric radiation therapy (RT). This study aimed to identify barriers and facilitators to HRQoL implementation in paediatric RT clinical practice and requirements for a digital PROM platform, from the perspectives of healthcare professionals.

**Method:**

Exploratory semi‐structured interviews were conducted with multidisciplinary clinicians from two hospitals providing care to paediatric RT patients. Interviews were transcribed verbatim, descriptively coded and analysed using content analysis. Consolidated Framework for Implementation Research (CFIR) was used as a theoretical framework for data collection, analysis and interpretation.

**Results:**

Nine interviews were held with nurses (*n* = 3), radiation therapists (*n* = 3), radiation oncology registrars (*n* = 2) and a consultant family therapist. Participants identified digital, clinical and child‐friendly features to inform platform development. All participants recognised the proposed digital platform to be of value by generating new information to support patient care. The perceived alignment with clinical workflows, potential to provide staff satisfaction and individual scope to act on PROM results were key facilitators. Clinical time pressures, transient staffing and reluctance for change were identified as potential barriers. Engagement of clinical staff and training in addressing psychosocial concerns were recommended to support clinical actioning of results and foster successful clinical uptake.

**Conclusion:**

This study used CFIR to systematically identify requirements for a digital platform and barriers to routine patient‐reported HRQoL collection in the paediatric RT setting. The facilitators and complexities of PROM implementation can inform platform development and future implementation strategies.

## Graphical abstract

This study examined the barriers and facilitators to incorporating health‐related quality of life (HRQoL) assessment into routine paediatric radiation therapy practice from the perspectives of healthcare professionals. Key requirements for a digital patient‐reported outcome measure platform and recommendations for clinical implementation were identified.

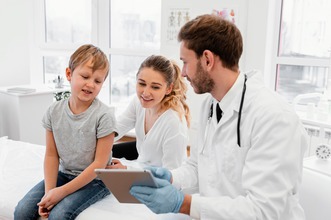

## 1 Introduction

Childhood cancer survivors are likely to experience substantial physical and/or psychosocial treatment‐related sequelae, which impact the child's health‐related quality of life (HRQoL) [1, 2, 3, 4]. In cohort studies of adult survivors of childhood cancer, 62% had at least one chronic health condition [5], and three in four survivors experienced a late effect of their cancer treatment [4]. Survivors who rate their health poorly also are likely to experience more symptoms of anxiety, depression and somatic distress [6, 7, 8], which significantly contribute to poor HRQoL [1, 3].

HRQoL is a multidimensional construct, defined as the individual's perception of their position in life as impacted by their health status [9, 10]. Due to the personal and subjective nature of HRQoL, patient‐reported outcome measures (PROMs) are used to gain experiential insights and direct reports from the patient and their caregiver about the child's HRQoL [11]. Use of PROMs in paediatric oncology has the potential to empower the child's voice, improve awareness of patient concerns, foster communication between clinicians and patients and improve symptomatic control [12, 13, 14].

Despite growing evidence, using PROMs to assess the impact of treatment or supportive therapies on HRQoL is rare in paediatric radiation therapy (RT) clinical practice [15, 16, 17, 18]. While RT is often an essential component of cancer treatment, it has been associated with a high burden of adverse events and chronic health conditions that impact HRQoL [4, 19, 20, 21]. Therefore, implementing patient‐reported HRQoL assessment in clinical practice is crucial to support and improve outcomes for paediatric RT patients.

Reported barriers to routine PROM use in radiation oncology include the administrative burden of paper‐based measures, perceived irrelevance when results do not inform care and unsustainable implementation due to a lack of alignment with existing clinical workflows [22, 23]. Digital adaptation of validated PROMs can maintain the reliability of paper‐based measures, increase engagement and overcome several barriers to implementation [24, 25, 26, 27]. Therefore, development of an electronic PROM platform for the paediatric RT setting has the potential to facilitate uptake of HRQoL evaluation in routine clinical practice. In this study, we aimed to: (1) identify operational requirements from paediatric RT healthcare professionals (HCPs) for a digital PROM platform to support routine HRQoL assessment and (2) identify the barriers and facilitators to implementation of the proposed digital platform in paediatric RT clinical practice.

## 2 Methods

The ethics statement was received from the Women's and Children's Hospital Human Research Ethics Committee (Application ID: 2021/HRE00094).

### 2.1 Study Design

This study is part of a larger project aiming to codesign a digital platform to support patient‐reported HRQoL outcome collection in paediatric radiation oncology. In this qualitative descriptive study, we describe the first component of the generative phase of codesign, understanding the needs and experiences of HCP stakeholders [28, 29].

**FIGURE 1:**
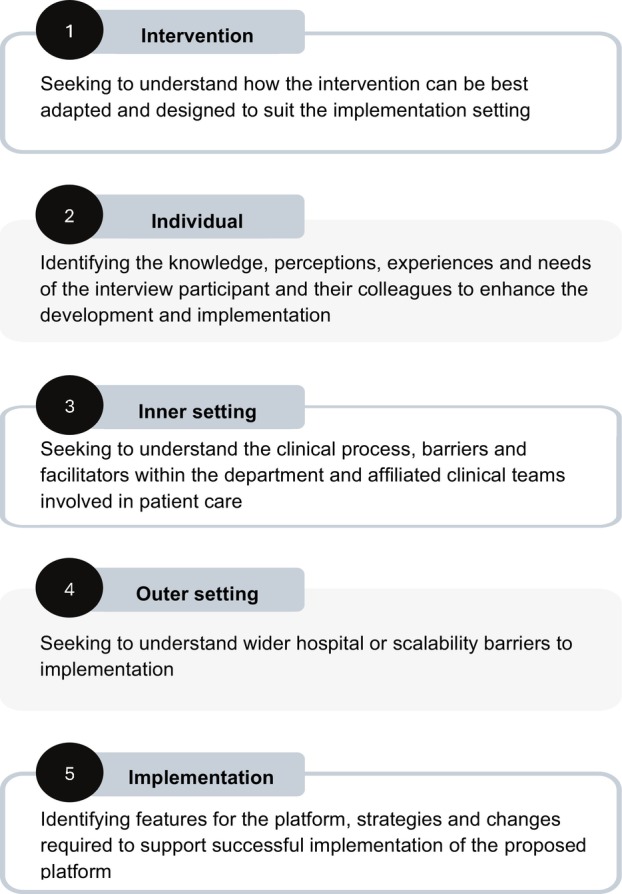
Consolidated Framework for Implementation Research (CFIR) domains and study application.

Consolidated Framework for Implementation Research (CFIR) was used in this preimplementation study to facilitate the design of the interview guide and inform the interpretation and reporting of results. CFIR is an implementation framework containing 39 standardised implementation‐related constructs that are organised across five domains, designed to facilitate systematic assessment of barriers, enablers and contextual determinants of implementation [30, 31]. Figure 1 shows the five CFIR domains and their application to the study context.

### 2.2 Study Participants

Eligible participants were HCPs with experience providing care to paediatric RT patients. All healthcare disciplines or specialities were eligible, including radiation oncology, paediatric oncology, nursing, allied health and social work professionals. HCPs were eligible if they provided care to patients at any stage of diagnosis, treatment or survivorship.

Recruitment commenced on 30 August 2021, at the Royal Adelaide Hospital, and 6 June 2022, at the Women's and Children's Hospital with an email to all potentially eligible staff groups. Open recruitment methods, including flyers in the hospital setting and two rounds of social media and email advertisements, were also used. Informed consent was obtained from all participants prior to their involvement in the study. Recruitment closed at both sites on 7 August 2022, after the final participant who had expressed interest was interviewed.

### 2.3 Procedure

Semi‐structured interviews using a naturalistic inquiry approach were conducted with eligible HCPs [32]. Interviews were conducted between September 2021 and August 2022. A semi‐structured interview guide (Supporting Information 1) was followed for all participants, with occasional prompts by the interviewers for clarification. Interviews were conducted online using Zoom (Zoom Video Communications Incorporated, San Jose, California, USA), or in person, based on the participant's preference and/or COVID‐19 restrictions at the time of the interview. Interviews were recorded using a Philips Voice Tracer DVT1000/00 digital voice recorder or via Zoom function and transcribed verbatim. Interviews were led by the senior author (MS), a senior researcher with qualitative research expertise, and supported by authors MD and AC who ensured adherence to the interview guide and provided further probing questions.

### 2.4 Patient‐Reported Outcome Measures

Participants who were not familiar with PROMs were shown self‐report and parent proxy report versions of Paediatric Quality of Life Inventory (PedsQL) Generic Core Score, Version 4.0, as an example during the interview. PedsQL is a validated 23‐item questionnaire, grouped into four key domains of physical, emotional, social and school functioning, with appropriate wording and response categories according to developmental stage [33]. PedsQL is the most used PROM in studies describing HRQoL outcomes for paediatric RT patients and has been used to inform clinical decision‐making in paediatric oncology [17, 18].

### 2.5 Data Analysis

NVivo (QSR International, 2022) was used to manage qualitative data. Content analysis with descriptive coding was performed. The first author (MD) followed content analysis preparation and organising phases [34]. As the semi‐structured interview questions were developed to correspond with constructs within CFIR domains, interview question categories were used for initial deductive coding of data. Inductive coding was then performed to identify patterns and regularities of emerging requirements, barriers and facilitators. Applying an iterative categorisation approach [35], the data were revisited repeatedly until no further codes emerged and all coding was consistently categorised within the CFIR constructs. Data were single coded by the first author (MD) and all final coding was reviewed by the senior author (MS) to ensure that the coding frame was applied consistently. Multiple codes that were separate in initial coding but fell under a broader umbrella of a single construct were unified through discussion with a third author, AH.

## 3 Results

Nine HCPs including radiation therapists (*n* = 3), radiation oncology registrars (*n* = 2), a radiation oncology nurse, a paediatric oncology nurse coordinator, a paediatric oncology nurse practitioner and a consultant family therapist were interviewed. Most participants were female (*n* = 7). The participants' length of experience working with paediatric oncology patients ranged between 2 and 20 years. The average interview length was 60 min (range: 45–92 min). Four interviews were conducted in person, and five were conducted online.

Data were grouped into the corresponding categories from the CFIR domains. Results were subcategorised within CFIR domains and the relevant corresponding constructs.

### 3.1 CFIR Domain 1—Intervention Characteristics

#### 3.1.1 Construct—Innovation Design

Findings for operational requirements of the proposed digital platform of PedsQL and supporting quotes are presented in Table 1.

**TABLE 1:** Platform requirements recommended by healthcare professionals.

	Requirement	Supporting quote
Child interface	Pictures to provide context or clarification	*‘Pictures are usually pretty good as well to help explain things and to make it clear… I'm talking about emotions, or how strong you are and healthy you are…’* [N2]
	Accessibility	*‘… some of our brain tumour patients might have some difficulties with [paper‐based questionnaire completion], so that's where you might have to rely on parents or consider things like Easy Read [to enable communication access for all abilities] [N2]* *Even having the questions audible because a lot of patients might not be able to read…’* [RT2]
	Emojis/smiley faces	*‘…would be good for the kids to answer using smiley faces or things… that are interactive…’* [RT2]
	Interactive engagement (gamification)	*‘…making it kid‐friendly so that they will engage with it’*. [N3]
	Ease of use	*‘I think keeping it simple is probably the best thing. You don't want to make it too complex when you're working with kids’*. [N2]
	Diversity and inclusivity	*‘…having it in multiple languages as well… just in terms of diversity, having the ability to have as many people feel like they can access it’*. [RT2]
	Sections for domains	*‘…when you see [the children] filling it out [paper‐based forms]… I don't feel like there's the thought that's gone in behind it. So, I think even just a break between [each domain]. A 10‐s clip that makes them reset’*. [N1]
Clinical interface	Clinical alert for clinically meaningful response	*‘I think trends would be really good… I'm just pulling random numbers, but if it's consistently sitting around a five beforehand then actually that's a two‐drop, that should be alerted’*. [N2]
	Clinical alert upon completion	*‘… if we were sent a notification to say the person's uploaded a document’* [RT2]
	Summary scores	*‘I think having a summary for each part, just for ease of reading would be useful for us’*. [RO1]
	Normative reference data	*‘… you would look at the answer and say where does that sit with most children going through treatment, I want to know where my child's sitting’*. [RT2]
	Comparison to previous own score	*‘I think a summary of what they are saying* versus *what it's been for the week before, because I know there is an element of most of us that look at where the patient's at before they come in…’* [N1]
	Easy and quick to use	*‘And you don't want it to be slow and lagging when you load something… you want it to be accessible and just going “oh cool I've got that information right there, I can easily see it”’*. [RT3]
	Ability to view and action results is essential	*‘I think the only thing that would put me off was if we weren't able to follow up on things that were highlighted… not actually being able to put into practice in making things better for kids and their families…’* [N3]
	Dashboard	*‘Something like a dashboard would be nice’*. [RO2]
	Search function	*‘…something that's easy to search would be ideal’*. [RO2]
	Clinical notes	*‘… somewhere to put notes or a comment about what was done about a particular assessment’*. [RO2]
Digital features	Electronic medical record (EMR) integration	*‘I think the easiest access point if we were to access it… would be embedded under documents in [EMR], so somewhere where everyone can access it… so it can be shared across other health professionals…’* [RT3]
	Simple PDF report	*‘I think with the software we're currently using and the problems already that we have with interoperability… a clean PDF would be the way to go’*. [RO2]
	Compatible on all devices	*‘I would say [available to use on] all devices…’* [RT1]
	Results in standalone platform	*‘…In a way, [results] in the platform would be better if there was access to it that you could generate the reports…’* [RT1]
	Maintain a paper‐based option for completion	*‘… some families will go “no we don't allow iPads”, but I would be quite happy to do this ‘cause I like paper (gesturing to PedsQL printout). Give them a choice…’* [T1]

Abbreviations: N, nurse; RO, radiation oncologist; RT, radiation therapist; T, family therapist.

Recommendations for the proposed platform were categorised into child interface, clinician interface and digital requirements. Frequent suggestions for the child interface included features to support engagement and the interpretation of questions by children. Other requirements included child‐friendly PROM completion and navigation, inclusion of multiple language options and diverse representation of children within the supporting imagery. Requirements for the clinical interface included methods to assist clinical interpretation, such as clinical alerts, summary scores, normative reference data and longitudinal trend visualisation.

#### 3.1.2 Constructs—Innovation Source (PedsQL) and Relative Advantage

Table 2 presents an overview of the barriers and facilitators to the implementation of the proposed digital platform, corresponding with CFIR constructs and domains.

**TABLE 2:** Barriers and facilitators identified using the consolidated framework for implementation research (CFIR).

CFIR domain	CFIR construct	Barriers & facilitators
Intervention	Innovation source and relative advantage	Reflection prompt, promotes child's voice
		Answering psychosocial questions may lead to feelings of distress
Individual	Knowledge & beliefs about the Innovation	Clinically advantageous, generates new information
	Self‐efficacy and individual state of change	Essential to clinical action results
		Time
Inner setting	Implementation climate	Reluctance for change
		Staff satisfaction, enhance information provision
		Potential method to standardise care
	Networks and communications	Email notification for clinically significant result
		Complexities in information provision to parents
Outer setting	Cosmopolitanism	Preplanning of distress protocol/referral pathway
		Promote communication between clinical teams
Implementation	Data collection	Provide families with options for PROM completion
	Suggested implementation strategies	Training
		Frequency of use
		Clinic champion

Abbreviations: CFIR, consolidated framework for implementation research; PROM, patient‐reported outcome measure.

Despite eight of nine participants having no experience using PedsQL, all participants mentioned that the selected measure would be useful to support clinical care. The preferred use of the acute (participants to respond considering the past 7 days) or general (participants to respond considering the past month) version of PedsQL varied, depending on the frequency of PROM collection, the individual patient's clinical status and the age of the child. Frequent device use by children and parents in the waiting room was identified by participants as a key facilitator to support patient uptake. A self‐reporting PROM was identified as a beneficial reflection prompt and a meaningful method to promote the child's voice, without fear of upsetting their family. *‘To have some honest feedback from the child I think would be so valuable because you know are they going to say I worry about what will happen to me in front of their Mum and Dad?’* [Nurse].

While the questions posed in the PedsQL PROM surrounding psychosocial health were viewed as important to assess, some participants raised concerns that answering psychosocial questions may bring up past feelings of distress for some children and/or parents, which could in turn affect implementation uptake. *‘I can only imagine [participation] would be dependent on how they're feeling through the treatment. So, they might not be up to it, or like these kinds of heavy questions, towards the middle and end (gesturing to emotional, social and school domains)’.* [Radiation Therapist].

### 3.2 CFIR Domain 2—Individual

#### 3.2.1 Construct: Knowledge and Beliefs About the Innovation

PROMs were not used by any of the interviewed staff in current clinical practice during active treatment or survivorship.

All participants expressed that they would like to use the proposed platform and that it should be used for both clinical and research applications. Implementation of HRQoL assessment using PedsQL was perceived by all participants to be clinically advantageous, as it could generate new information to guide patient management. Some participants felt that aspects of psychosocial health are not systematically assessed in current practice and routine use of the proposed intervention could provide a solution. One participant mentioned that *‘everybody gets so focused on physical… their biggest issues aren't physical, their biggest issues are actually mental health… I think it [HRQoL collection] should be part of standard practice and I think there's no reason why you couldn't be doing [the PROM] prior to each of your reviews.’* [Nurse].

#### 3.2.2 Constructs: Self‐Efficacy and Individual State of Change

There was consensus that it was within the scope of the participant's own practice to act on an HRQoL report of concern and that HCPs would have the clinical capacity to do so. The key motivation for the use of PROMs was the ability to further enhance the clinical care of patients. The need for and importance of clinically actioning the HRQoL results was strongly emphasised by most participants. *‘…the only thing that would put me off was if we weren't able to follow up on things that were highlighted… just doing this as a tick box exercise but not actually being able to put in to practice, in making things better for kids and their families…’* [Nurse].

The proposed platform was perceived as complementary to current standards of care and deemed by participants that it could *‘…be very easily incorporated into processes that already exist in the department’*. [Radiation Therapist], with all participants suggesting timings to incorporate PROM collection and evaluation into existing clinical tasks. One HCP mentioned their own reluctance to use a new digital tool based on their perceived confidence in using technology, as a potential barrier to implementation. All other participants only perceived the use of a digital platform as a barrier if the system were to become slow and require additional clinical time. Incorporation of easy‐to‐view summaries to facilitate clinical interpretation was suggested to provide HCPs with a *‘one‐stop shop’* [Nurse] that may allow a reduction in clinical time by directing the existing clinical review according to areas identified in the PROM.

### 3.3 CFIR Domain 3—Inner Setting

#### 3.3.1 Construct: Implementation Climate

Many perceived that the proposed intervention would provide staff satisfaction through the visualisation of individual patient outcomes across their treatment journey, to understand the impact of their care provision and to generate knowledge to enhance the information provision of the follow‐up experience for future patients.

Reluctance for change, due to the lack of available clinical time, was the key perceived barrier to implementation with the participant's colleagues. This was often presented with the caveat that if the benefits to patients and clinical systems were presented, their colleagues would be motivated to participate. *‘People might be… reluctant. They might think “I've got to already do so many things and I'm not adding this in”. But I guess if it's spelled out to them the benefits for why… I can't foresee why they [clinical staff] would think it was not beneficial for our ability to create better care for them [the patients].’* [Radiation Therapist].

The importance of ensuring clinical information and clinical alerts were accurate, relevant and received by the correct staff member was emphasised to support staff engagement with the proposed intervention. Transient staffing was identified as a complexity to implementation. However, some participants felt that the platform could be a beneficial tool to standardise care within transient staffing and shared‐care management processes.

#### 3.3.2 Construct: Networks and Communications

All participants agreed that the best point of contact for a clinically significant HRQoL report was an email to HCPs. However, this suggestion was offered with reluctance due to existing clinical systems already requiring frequent email notifications.

The sensitivities associated with parents receiving HRQoL results were identified as a complexity for implementation. Participants reported that while it was the correct duty of care to provide parents with access to the child's HRQoL outcomes, the manner in which these results should be managed and presented to parents should be sensitive, to protect the emotional well‐being of children and parents. One participant raised the complexity of navigating unsafe family dynamics or complex social situations, while several participants perceived that children may not truthfully self‐report the PROM questions if the children are aware of their parents viewing the results automatically on completion. *‘… children are very good at… hiding things from their Mum and Dad, and I think that's what concerns me is that, how honest are we going to get them to be, knowing that their Mum and Dad will know’.* [Nurse].

### 3.4 CFIR Domain 4—Outer Setting

#### 3.4.1 Construct: Cosmopolitanism

The interconnectivity between the two hospital sites and clinical teams supporting children with cancer was a perceived facilitator to implementation. The existing interconnectivity could support referrals or shared care that may be required based on PROM results, and promote scalability within all paediatric, adolescent and young adult oncology services. Participants identified that while this is a possibility, the referral process needed to be explicitly identified and planned prior to implementation. *‘I think we need to be realistic about if we get a kid scoring high (inferring at‐risk)… what do we do about that… what is the pathway for that?’* [Nurse].

Radiation oncology participants identified that they lacked awareness of the patient's follow‐up and survivorship management posttreatment by the child's primary care team. Some participants perceived the proposed intervention as a method to promote this communication and awareness between clinical teams. ‘*[it is difficult] knowing that there's the long‐term effects of radiotherapy and not knowing if it's being managed appropriately, or if they're [the patient is] getting the correct information. If there is some form of a shared platform that we can all access and retrieve information from, it would be amazing.’* [Radiation Therapist].

A desire to integrate the proposed intervention with electronic medical record systems was raised by most participants. However, the barrier of scalability was identified, as a centralised medical record system has been proposed but is not yet implemented across the two hospital sites involved in this study.

### 3.5 CFIR Domain 5—Implementation Process

#### 3.5.1 Construct: Data Collection

Ideal patient completion of the PROM using the platform was in the waiting room on a hospital‐supplied portable device or at home prior to a scheduled appointment. Participants suggested that both options should be presented, due to different patient preferences and habits. Participants stated that meaningful time points for PROM collection include baseline prior to any therapeutic intervention commencing, prior to weekly review appointments whilst undergoing active radiation therapy, and before follow‐up appointments.

#### 3.5.2 Construct: Suggested Implementation Strategies

Staff awareness of the intervention prior to implementation was suggested by all participants as a key strategy to support successful uptake. Providing training and instructional information materials was highly recommended as a facilitator. *‘I think you probably need to do training, just because obviously when you introduce any new tool, people should know what it's about. And I think you'd have to make it something that was consistent in practice as well.’* [Nurse].

Successful implementation and uptake of the proposed platform was perceived to be dependent on the frequency of use *‘… if it becomes outside of the routine, then that's when I think it would stop getting used.’* [Radiation Therapist], and the interplay of use between family and clinician consumer groups. For example, if the intervention is consistently used by HCPs, it will be used by families and vice versa. A clinic champion was suggested for each site to encourage and support implementation, *‘having someone who is the champion of this platform in our department would probably be ideal…’* [Radiation Oncologist].

## 4 Discussion

Despite the availability of PROMs to effectively capture HRQoL outcomes for paediatric patients, their use in paediatric RT clinical practice remains rare. In this study, we identified requirements for a digital PROM platform to support patient‐reported HRQoL data collection in this setting, along with perceived barriers and facilitators to successful implementation.

Key platform requirements included a child‐friendly interface, clinical interface and general digital functionalities. Many of the requirements align with known constructs for quality e‐health interventions including visual design, usability, user engagement and content qualities [36, 37, 38]. Participants emphasised the importance of child‐friendly visual design considerations, such as pictures and faces. Usability was highlighted as a key consideration, with participants specifying the need for easy navigation and a quick user experience to promote engagement among children, parents and clinicians. Additionally, clinical interface content requirements to assist interpretation were described, such as instantaneous summary scores, alerts for clinically meaningful results and comparisons to HRQoL norms. Broader principles, such as privacy and security, workload impact and user education [36, 38], were also highlighted by participants as critical considerations for clinical implementation.

A key facilitator identified the unanimous perception that the proposed intervention would be beneficial to support clinical management and was within their scope of practice. All participants highlighted the potential for PROMs to capture meaningful clinical insights that might otherwise go unnoticed. The primary motivation for use was the ability to enhance care provision, by actioning the HRQoL reports of current patients and by identifying trends to inform care of future patients. These findings are consistent with the perspectives of paediatric oncology professionals collected in an international survey [39], emphasising the importance of fostering a positive perception of PROM utility to drive adoption.

Additional facilitators included the existing frequent device use by children, parents and HCPs and the perceived alignment of PROMs with existing clinical workflows. However, the key perceived barrier was reluctance to change if the platform or its processes required additional clinical time. Integrating the platform into clinical workflows to minimise the additional workload required is a recognised enabler of PROM adoption in the oncology setting [22, 23]. As HCPs' perceptions of PROMs can directly influence uptake and acceptability [22], seamless integration with standard workflows will be critical to promote successful implementation and avoid burdening clinical staff with additional tasks.

Ethical considerations specific to the psychosocial domains of HRQoL assessment in the paediatric health context were also identified. Some HCPs perceived that responding to psychosocial functioning questions may be distressing for some children and parents. As participants reported that psychosocial health is not consistently assessed in a standardised manner for paediatric patients, clinician discomfort may also arise from a lack of experience in addressing these concerns. Training and education should be provided to support HCPs to review, action and provide care based on the reported outcomes [40, 41]. McCabe et al. (2023) recommend that clinical staff receive education on the PROM constructs, questions and suitability, to ensure measures are used and interpreted as intended [40]. HCP awareness of these items, alongside the use of validated PROMs with established appropriateness for the paediatric oncology population, will be important considerations to mitigate potential distress for children and caregivers.

Another complexity raised was the appropriateness of parental access to the child's self‐reported PROM results. While previous studies using PROMs in the paediatric setting have provided children and parents with access to the child's PROM results [42], the suitability of a child to keep their responses private from their caregivers depends on factors such as the child's age, national consent models, institutional policies and departmental preferences. This area should be explored further prior to the implementation of a digital PROM platform in Australia and will be particularly important for children approaching adolescence or who are deemed ‘Gillick competent’ [43].

A strength of this study was its use of CFIR to systematically assess the implementation setting, identifying barriers and facilitators specific to paediatric RT practice. The use of an implementation science framework to comprehensively categorise barriers will promote the use of enablers in the platform development and will support the identification of appropriate Expert Recommendations for Implementing Change (ERIC) implementation strategies to inform future phases of this research [44]. Limitations include the small sample size and potential self‐selection bias among participants who showed interest in participating, as they may have had a preexisting interest in PROMs. Additionally, this study only presents the views of health professionals and does not report the perspectives of patients or caregivers. Future research should prioritise and explore the perspectives of children and parents, including engagement with consumers as partners to codesign the digital PROM platform.

## 5 Conclusion

Routine collection of HRQoL outcomes in paediatric RT clinical practice is rare despite the potential to enhance patient care. This study identified operational requirements to inform the development of a digital PROM platform to support routine HRQoL assessment in paediatric RT. The perceived barriers and facilitators to the implementation of PROMs were identified and can be used to inform PROM implementation strategies in paediatric RT and similar clinical settings.

## Conflicts of Interest

The authors declare no conflicts of interest.

## Supporting information


**File S1.** Contains interview questions from the moderator guide.

## Data Availability

The datasets generated and/or analysed during the current study are not publicly available due to ethical considerations but are available from the corresponding author on reasonable request.
