# Supplementary material for: Healthcare Professional Perspectives on Digital Health‐Related Quality‐of‐Life Assessment in Paediatric Radiation Therapy: A Qualitative Study
Source: J Med Radiat Sci. 2025 Apr 28;73(Suppl 2):S46–54. doi: 10.1002/jmrs.880 (PMC13121558; doi:10.1002/jmrs.880)
Supplement: Supplementary file 1 — File S1. Contains interview questions from the moderator guide. [file JMRS-73-S46-s001.docx]

**Supporting Information 1- Interview Questions from Moderator Guide**

**Introduction**

*Phrase questions based on clinician’s role*:

**Initial demographic/icebreaker questions**

1. Tell us about your current and previous roles working with paediatric radiation therapy patients.
2. What does your role(s) entail?
3. How many years of experience do you have working with children with cancer?

**During treatment processes**

1. How often do patients with childhood cancer attend review during radiation therapy?
2. Do you collect patient reported outcomes (PROs) or assess health-related quality of life (HRQoL) for paediatric patients whilst they are undergoing radiation therapy?
   - If so what tools do you use? What outcomes are assessed?
   - If yes, do you find it useful? Does it inform clinical decisions?
3. Do you collect PROs or assess HRQoL for any other patient group during treatment?
   - If yes, how is this similar to your paediatric workflow?

**Follow up processes**

1. What is the current workflow for follow up of paediatric patients? Do paediatric patients attend this department for long-term follow-up?
2. Do you collect PROs or assess HRQoL for paediatric patients after treatment?
   - If so, what tools do you use? What outcomes are assessed?
   - If yes, do you find it useful? Does it inform clinical decisions?

**Example - PedsQL**

Show printout PedsQL. Explain PROMs using PedsQL example of child self-report and parent-proxy report for those who are not aware. This may have already occurred in response to previous questions or conversation.

1. Have you used PedsQL or other HRQoL PROMs before?
   1. If yes, tell us about your experience. What worked, what didn’t?
2. By looking at the PedsQL example print out, what window of the patient recalling back their HRQoL would be most useful for you to interpret?
   (Last 7 days or month)
   1. Why?
   2. Does the clinical relevance differ based on the patients’ time from treatment?

**I****deal features**

1. Do your patients use any e-health tools during or post-treatment?
   1. Parent tools? Child tools?
   2. If yes, is there anything that would be ideal to use in our proposed platform?
2. What key design features should the platform include?
3. What features should NOT be included?
4. What device would you like to use the platform on?
5. How would you like to receive the results if this platform was implemented?
   1. In what format?

**Clinical Implementation**

1. How would collection of HRQoL best be implemented within your current workflow?
2. What tasks do you already complete that you can envisage that collecting PROMs with this proposed platform could compliment?
3. If this platform was implemented within this patient group, what would be the most useful features for you?
4. If this platform was implemented within this patient group, when is the optimal timing of completion?
5. Would you like to be notified of patient/parent completion?
6. Would you like the parent to be notified if there was a ‘clinically significant’ decline in results from the previous result?
   1. Probe for why/why not
7. Would you (as the clinician) like to be notified if there was a ‘clinically significant’ decline in results from the previous result?
   1. In addition to or instead of the parent notification?
   2. If yes, how should the notification occur?
8. Do you have the clinical capacity to act on severe report?
   1. Would you only use proposed platform prior to a scheduled appointment?

**Barriers and facilitators to implementation**

1. What is your view on how the information collected is used? Should it be used for research only (data collection and retrospective analysis) or clinical or both?
2. Do you think collecting PROMs to assess HRQoL is useful to assist clinical decision making in this population?
   1. Why / Why not?
   2. When would it be the most useful?
3. Can you talk us through what resources you would need to use the platform?
4. What factors are essential in successfully implementing this into routine care, and why?
   1. At a systems level?
   2. At an individual level?
5. Do you see yourself using the platform?
   1. Why or why not?
6. What are your thoughts on patients and their parent/caregiver being able to use this proposed platform on a regular basis in the long term?
7. How do you think others (add examples based on clinical role/site) would find using the platform regularly?
   1. Why do you think that’s the case?
8. What would stop you from using the platform and why?
9. What do you foresee being the barriers to patient utilisation?

**Conclusion**

Questions from participant

Information about next steps in study
